# Which Country is Truly Developed? COVID-19 has Answered the Question

**DOI:** 10.5334/aogh.2894

**Published:** 2020-05-18

**Authors:** Jeffrey S. Freed, Soo Y. Kwon, Hannah Jacobs El, Michael Gottlieb, Ram Roth

**Affiliations:** 1 Department of Surgery, Icahn School of Medicine, New York City, US; 2 Department of Obstetrics and Gynecology, Zucker School of Medicine at Hofstra, Northwell Health, New York City, US; 3 Department of Epidemiology, Columbia University, Mailman School of Public Health, New York City, US; 4 Foundation for the National Institutes of Health, Bethesda, Maryland, US; 5 Department of Anesthesiology, Perioperative, and Pain Medicine, Icahn School of Medicine at Mount Sinai, New York City, US

## Abstract

The developed countries of the world were ill-prepared for the pandemic that they have suffered. When we compare developed to developing countries, the sophisticated parameters we use do not necessarily address the weaknesses in the healthcare systems of developed countries that make them susceptible to crises like the present pandemic. We strongly suggest that better preparation for such events is necessary for a country to be considered developed.

The health of a country’s population is directly correlated with increased life expectancy and productivity, as well as with its economic progress and ever-expanding wealth. The citizens of high-income “developed” countries, as defined below, depend on an advanced healthcare system as the foundation of the well-being of its citizens. Whether health care is provided by the public and/or by the private sector, delivery of care and managing disease are largely dependent upon public health resources that can identify and quantify disease threats at population levels. One may ask if a country’s public health system, even in high-income countries, is sufficiently equipped and prepared to address novel disease threats such as posed by infectious disease pandemics.

The ability of a country to support public health management services depends on coordination of multiple components including government health agencies at the central and local levels, donor organizations, civil society groups, and directly with affected communities. These relationships are meant to provide the services necessary for successful healthcare, but must also be responsible for planning for future needs and for executing programs to recognize and deal with emergencies. The coronavirus pandemic highlights the latter function as a determinant of which nations are adequately resourced and which have yet to achieve that status.

We have used multiple methods to determine which countries are to be accepted as developed [1]. Gross domestic product (GDP) per capita, a compendium (in US $) of all the goods and services produced in a country per year, divided by its population, has traditionally, if unofficially, been used to determine whether a country is developing or has reached developed status [2,3]. One unofficial threshold for a country with a developed economy is a GDP per capita of $12,000 [4]. There is, of course, controversy regarding what economists consider the threshold for consideration as a developed country. Many economists prefer using a per capita GDP of at least $25,000 for a country to be considered developed. In 2018, the gross domestic product per capita in the United States amounted to approximately 62,868 U.S. dollars [5].

Exceeding even the $12,000 GDP is not an absolute entry ticket to developed status. There are other criteria created by different international agencies that developed countries must meet. For example, the World Development Indicators (WDI) are the World Bank development indicators collected from officially recognized international sources. It presents what is considered the most up to date and accurate global development data and includes national, regional, and global estimates. Also, the United Nation Development Programme Human Development Index (HDI) is often used to classify a specific country. The HDI is a compilation of average achievement in what is considered most important measures of human development: a longevity and health, intellect, and community-wide acceptable standard of living. The HDI is a statistical methodology that normalizes the indices (geometric mean) associated with each of these three major quality-of-life areas.

A developed country is defined, according to the United Nations Developed Country List (2020), as a sovereign state that has a developed economy and technologically advanced infrastructure when compared to other nations [6]. According to this definition, several factors determine whether or not a country is developed, such as the Human Development Index (HDI), political stability, gross domestic product (GDP), industrialization, and freedom. As stated above, the healthcare systems of the countries considered developed would play a significant role in many of these factors.

Based on such rankings created by these knowledgeable agencies, one can make the argument that more developed countries are better able to provide public health resources (e.g. surveillance systems, epidemiological models, clinical trial networks to test interventions, etc.) that form the basis for quantifying disease and for establishing policies and research priorities for dealing with such recognized disease threats. In contrast, under-resourced countries are usually ill prepared and lack the health management tools to deal with ongoing disease threats, even those for which interventions such as proper sanitation, vaccine, and therapeutics are already available.

However, even in “developed” countries, the lack of appreciation of the importance of planning for health and humanitarian emergencies was spotlighted as the European refugee crisis deteriorated during the last two decades. Mass movement from the so-called developing world into European Union countries was a reminder of glaring global inequalities and the lack of preparation of the so-called developed countries for this deluge of refugees with multiple needs, including that of healthcare. And this was only the beginning of the stresses on the developed nations of the world.

Currently, a devastating pandemic, attacking essentially all of the “developed” countries, has unmasked their lack of readiness for this moment. Unlike previous crises, this event has not been limited to the developing countries (e.g. the Ebola epidemic in West Africa or the tsunami in Indonesia). The present healthcare crisis has been ravaging the globe in an unremitting and deadly fashion. Lack of preparedness was exposed in a way that not only revealed the failure to consider this possibility, but also exposed our lack of technical capability to coordinate our efforts nationally and internationally. These shortcomings have led to an increase in the number of cases, the number of deaths, and delay of coordination and cooperation necessary to contain the pandemic.

This pandemic of 2019–2020 (to date) has exposed weaknesses, including lack of national and international cooperation, even among the most developed nations. These weaknesses included the lack of understanding of the virulence of such a virus as COVID-19, its asymptomatic phase capability of transmission, and its ease of transmission. Most importantly, it exposed our lack of consideration of the very possibility of a catastrophic global healthcare crisis. Developed countries had not considered how testing would be developed, how transparent reporting would take place, or how a vaccine would be developed. In view of these facts, all the nations of the world must be considered developing nations at present. We cannot depend on our GDP or our HDI to determine who is advanced and who is not. That is the lesson of the global coronavirus pandemic.

The take away is we are only one airline flight away from the next pandemic. To the COVID-19 virus, it does not matter if a nation’s GDP is high. GDP and HDI do not make citizens immune to such pandemic viruses. Creating better global public health infrastructure will well affect the health of citizens in every country of the world, regardless of wealth status on a United Nations or World Bank list.

The world healthcare system must be vigilant and responsive to any “hotspot” in any developed or developing country. Preparation and consideration, both nationally and internationally, must be our mantra. The failure to do so is an invitation for COVID-19, or any other virulent plague, to visit us again.

“Prior preparation and planning prevents poor performance” (author unknown).
